# Transforming nursing education: the power of educational leadership in optimizing time management and competency

**DOI:** 10.1186/s12912-025-03420-2

**Published:** 2025-07-07

**Authors:** Shaimaa Mohamed Amin, Ahmed Abdelwahab Ibrahim El-Sayed, Ahmed Abdellah Othman, Ahmed Salah Ali, Nagwa Ibrahim ELfeshawy, Hanan Hosni El-Sherbini, Abeer Abdel Fattah Mahmoud, Mohamed Hussein Ramadan Atta

**Affiliations:** 1 Community Health Nursing Department, Faculty of Nursing, Damanhour University, Damanhour City, Egypt; 2 Nursing Administration Department, Faculty of Nursing, Alexandria University, Alexandria, Egypt; 3 Lecturer of Nursing Administration, Faculty of Nursing, Sohag University, Sohag City, Egypt; 4 Pediatric Nursing, Faculty of Nursing, Mansoura University, Mansoura, Egypt; 5 Woman’s Health and Midwifery Nursing, Faculty of Nursing, Mansoura University, Mnansoura City, Egypt; 6 Maternal and pediatric Nursing, College of Nursing, King Khalid University, Abha, Saudi Arabia; 7 Community Health Nursing Department, Faculty of Nursing, Alexandria University, Alexandria City, Egypt; 8 Nursing Education Department, Faculty of Nursing, Damanhour University, Damanhour City, Egypt; 9 Nursing Department, College of Applied Medical Sciences, Prince Sattam Bin Abdulaziz University, Wadi Addawasir, Saudi Arabia; 10 Psychiatric and Mental Health Nursing Department, Faculty of Nursing, Alexandria University, Alexandria City, Egypt

**Keywords:** Time management, Nursing competence, Educational leadership, Nursing students, Mediation, Egypt

## Abstract

**Background:**

Effective time management and nursing competence are crucial for nursing students’ academic and professional success. Educational leadership may play a mediating role in strengthening the relationship between time management skills and nursing competence. However, limited research has examined this potential mediation in the nursing education context in Egypt.

**Aim:**

To investigate the mediating role of educational leadership in the relationship between time management and nursing competence among undergraduate nursing students.

**Methods:**

A descriptive comparative cross-sectional study was conducted, involving a sample of 532 undergraduate nursing students selected using systematic random sampling. Data collection tools included the Educational Leadership Scale for Nursing Students, the Nurse Competence Scale, and the Student Time Management Scale. All instruments were translated into Arabic and validated for the study population. Data were analyzed using correlation and mediation analysis to assess the relationships between variables.

**Results:**

The study revealed that nursing students’ educational leadership is strongly associated with both their competencies (*r* = .504, *P* < .001) and time management skills (*r* = .238, *P* < .001), while competencies also showed a moderate positive correlation with time management (*r* = .394, *P* < .001). Age, academic year, and family income were significantly associated with differences in educational leadership, competencies, and time management. Moreover, structural equation modeling showed that time management had a significant direct effect on both educational leadership (B = 0.411, *P* < .001) and competencies (B = 0.024, *P* < .001), while educational leadership significantly influenced competencies (B = 0.48, *P* < .001). Importantly, educational leadership mediated the indirect effect of time management on competencies (B = 0.567, *P* < .001).

**Conclusion:**

Educational leadership plays a mediating role in the relationship between time management and nursing competence, highlighting the importance of leadership development in nursing education programs. Nursing curricula should incorporate leadership training to improve students’ time management skills and competencies, ultimately enhancing their preparedness for professional practice.

**Implication:**

Nursing curricula should prioritize the integration of leadership development and time management skills into both theoretical coursework and clinical practice. To achieve this, actionable leadership training strategies could include the implementation of structured leadership workshops and seminars that focus on key competencies such as communication, decision-making, conflict resolution, and team management.

**Clinical trial number:**

Not applicable.

## Introduction

Time management is crucial for nurses as it directly impacts their ability to navigate role conflict and maintain work-life balance, ultimately affecting their competency in providing quality nursing care. In a demanding healthcare environment, nurses often juggle multiple responsibilities, such as patient care, administrative duties, and collaboration with multidisciplinary teams [1].

Effective time management allows nurses to prioritize tasks, allocate appropriate time for patient interactions, and reduce stress associated with overwhelming workloads. This balance is essential for sustaining their professional efficacy and preventing burnout, which can detract from their capacity to deliver optimal patient care [2]. An Egyptian study emphasized the negative relationship between internet addiction and time management among undergraduate nursing students [3]. Furthermore, continuous education, particularly in educational leadership, enhances nurses’ time management skills by equipping them with strategies to organize their work and make informed decisions effectively [4]. A recent Egyptian study emphasizes the role of career practice, in enhancing nursing Competency [5]. Such training fosters a culture of accountability and efficiency, enabling nurses to refine their competencies and adapt to the evolving healthcare landscape [6, 7]. By investing in continuous education and leadership development, healthcare organizations can empower nurses to enhance their time management abilities, improving nursing care competency and patient outcomes [8].

In the Egyptian context, these issues are particularly significant due to the unique challenges faced by the country’s healthcare system, including high patient loads, limited staffing resources, and variable access to professional development opportunities. Therefore, by investing in contextually relevant continuous education and leadership development, healthcare organizations in Egypt can empower nurses to enhance their time management abilities, improving nursing care competency and patient outcomes [5, 8].

Educational leadership is a crucial aspect of nursing, as modern nursing requires a wide range of knowledge and skills. While traditional nursing primarily emphasizes providing care and comfort, today’s nurses take on diverse roles, including caregiver, decision-maker, patient advocate, manager, rehabilitator, communicator, educator, and empowered [9, 10]. Educational leadership abilities can be acquired and refined over time, and it is essential to begin learning these skills early in a nurse’s academic journey [11, 12]. Many researchers emphasize that nurses must discern when to use specific methods and possess the necessary theoretical knowledge and practical expertise to apply them effectively [13–15]. According to the educational leadership literature, effective leadership emerges through scientific, educational, and visionary guidance [16, 17]. Nurse educators, therefore, are responsible for providing students with essential theoretical knowledge and practical experiences while fostering a lifelong desire for continued learning [9].

Time management encompasses a range of behaviors aimed at effectively organizing and allocating time [18]. These behaviors facilitate improved time utilization and productivity, ultimately increasing the chances of reaching set objectives [19]. Critical components of time management include developing skills in goal setting, prioritization, planning, and identifying strategies to minimize time wastage [20]. This skill set is vital for individuals in high-demand positions, such as nursing, where professionals face significant daily workloads [21]. The combination of heavy workloads, time constraints, and the necessity to make swift decisions underscores the importance of time management skills in nursing practice [19, 22].

Nurses consistently face the challenge of demonstrating their contributions to society as professionals. They are expected to assume responsibility for delivering ongoing direct care, safeguarding individual lives, and assisting with daily living activities, which may alter their altruistic nursing role [23]. In their practice, nurses must utilize their acquired knowledge, skills, and inherent personal qualities to address various situations, adapting their expertise to meet different needs [15]. Nursing competency is “the ability to take action by combining knowledge, skills, values, beliefs, and experience acquired as a nurse”. They emphasized that competency should be understood as an integrated performance that reflects the professional nurse’s emotions, thoughts, and decision-making abilities [24].

Effective time management directly fosters a productive learning environment and ensures high-quality patient care [25]. Educational leaders prioritizing time management can optimize scheduling, allocate resources efficiently, and create a structured framework supporting faculty and students [26]. This structured approach allows nursing educators to focus on developing essential competencies in their students, such as critical thinking, clinical skills, and teamwork. Additionally, nurses who master time management skills are better equipped to juggle patient care demands, administrative responsibilities, and continuous professional development, ultimately leading to improved patient safety and quality of care outcomes [27].

The literature findings identified four critical competencies in nursing education: articulating and promoting a vision for the field, serving as stewards of the organization and nursing education, upholding professional values within higher education, and developing nurturing relationships [28]. Effective time management significantly impacts nursing competence, particularly in delivering quality patient care shown that Time management training enables nurses to allocate and prioritize the time needed for various tasks [27, 29]. Furthermore, research indicates that nurses who participated in time management courses demonstrated superior time management skills compared to those who did not [30].

This gap highlights the need for further research on the relationship between educational leadership, time management, and nursing competency. Investigating these variables could provide valuable insights and contribute novel findings to the nursing field, particularly regarding how leadership education can be structured to enhance time management skills and, in turn, improve nursing competencies. Such research can empower nurse managers to develop future leadership education programs that foster positive work environments and enhance clinical outcomes [31].

The practical significance of the mediation findings lies in demonstrating that educational leadership is a key mechanism through which time management influences nursing competency. While time management alone positively affects competency, the presence of strong educational leadership amplifies this effect. In practical terms, this means that simply teaching time management skills may not be sufficient to maximize students’ clinical competencies. Instead, incorporating educational leadership training, which includes critical thinking, decision-making, and motivational strategies, can enhance the application and impact of those time management skills in clinical practice. Thus, the mediation effect emphasizes that leadership is not just a complementary skill but a transformative factor that shapes how foundational skills like time management translate into professional competence.

## Method

### Study design and setting

This research utilized a descriptive comparative cross-sectional design, guided by the Strengthening the Reporting of Observational Studies in Epidemiology (STROBE) checklist. This design was appropriate for comparing nursing students’ time management skills and perceived competencies across different academic levels and genders, allowing for the identification of variations between naturally occurring subgroups without manipulation of variables. The study was conducted at the College of Nursing, Sohag University, located in Sohag Governorate, Egypt. Sohag University was selected due to its status as a prominent public institution in Upper Egypt that enrolls a diverse nursing student population from various backgrounds. The college comprises nine specialized scientific departments, each representing a distinct nursing discipline. Both undergraduate and graduate programs operate under a credit hour system, providing a structured framework for tracking academic progress and enabling a comprehensive evaluation of educational outcomes.

### Sampling and study participants

The target population for this study was undergraduate nursing students enrolled in the faculty of nursing at Sohag University during the academic year 2024–2025. The inclusion criteria focused on undergraduate nursing students from all academic years. Participants must have completed at least one semester in their current year and provided informed consent. Additionally, they needed access to relevant resources for time management and competency development within their academic environment. First-semester students were excluded as they were still in the process of adjusting to university life and academic routines, which may not provide a stable or representative context for assessing their time management skills or competency perceptions. Furthermore, students with prior educational leadership roles were excluded to minimize bias that could influence their views on leadership and time management.

**Fig. 1:**
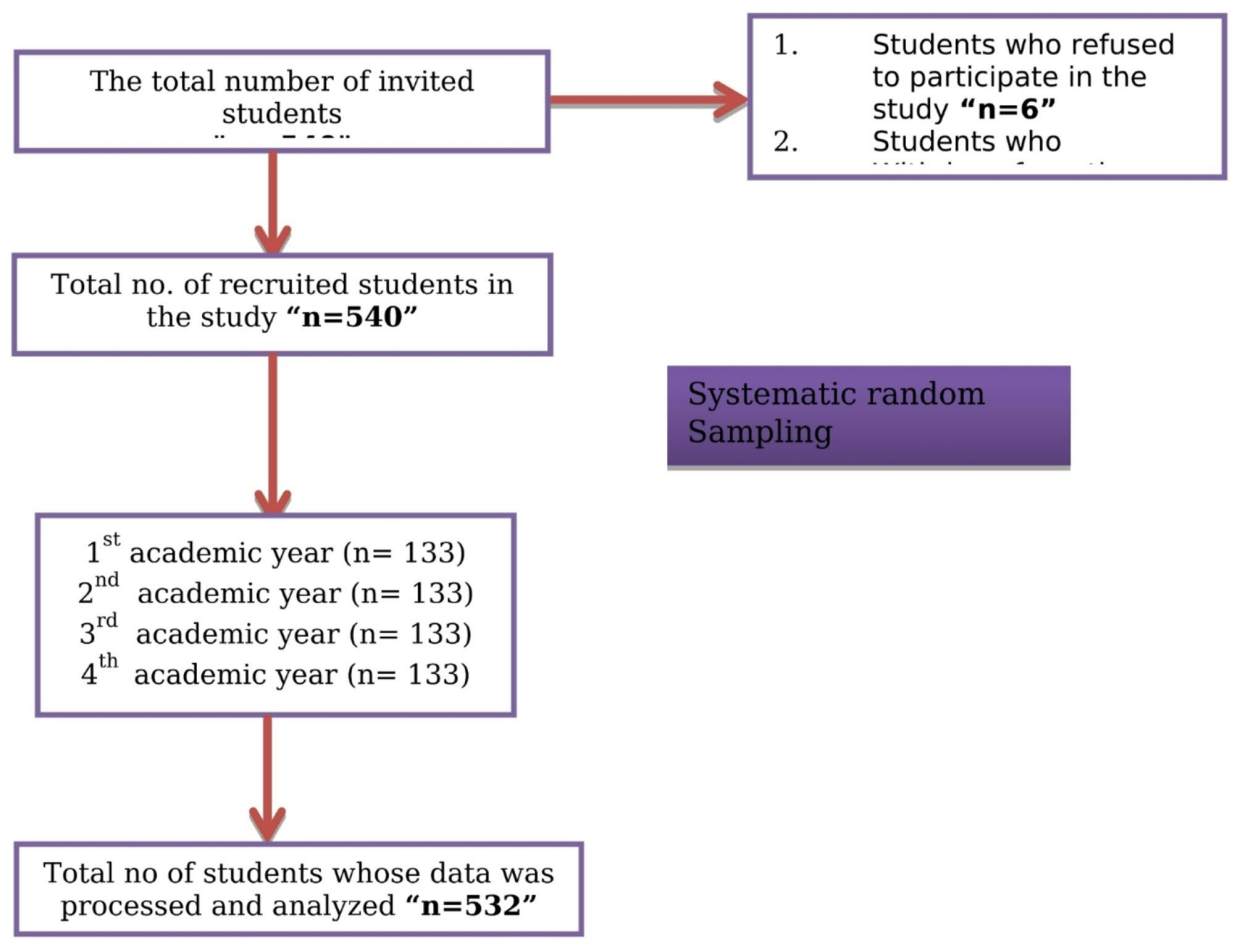
Flow chart of participants’ recruitment

G*Power software was used to calculate the study’s sample size, with 528 nursing students required to attain 0.98 power with a ratio of 1, an effect size f^2^ = 0.04, a total of 10 predictors, and two tested predictors for multiple linear regression analysis. It was upgraded to 540 to compensate for possible non-response. The final sample size for this study, consisting of participants who consented and completed the questionnaire, was 532. To ensure a balanced representation across all academic years, 133 students were selected from each cohort using an equal allocation method. Systematic random sampling was employed, where every 30th student on the list or within the population was chosen in a predetermined sequence, as illustrated in Fig. 1.

### Measurements of interest

#### Demographic form

The demographic form was developed by the researchers after reading the recent literature [32–34]. It included data such as age, sex, occupation, place of residence, family type, family income, and parents’ level of education.

### Educational leadership scale for nursing students

The Educational Leadership Scale for Nursing Students, developed by Karaman et al. (2023), was selected to assess nursing students’ leadership qualities and abilities within academic settings [8]. The scale includes 19 items that are divided into three subscales: Visionary Leadership (10 items), Instructional Leadership (5 items), and Scientific Leadership (4 items). Each item is rated on a five-point Likert scale, where 1 denotes “Never Disagree” and 5 indicates “Strongly Agree,” yielding a total score range from 19 to 95. A higher score on the scale reflects stronger leadership qualities. To interpret these scores, they were categorized into three levels: low (19–44), medium (45–69), and high (70–95). These classifications align with existing literature on educational leadership and provide a meaningful way to distinguish varying levels of leadership capability among students. In the present study, the Arabic-translated version of the scale demonstrated excellent reliability, with a Cronbach’s alpha of 0.94. The content validity of the scale was confirmed through exploratory factor analysis, which explained 75% of the total variance. Both the Kaiser-Meyer-Olkin (KMO) measure and Bartlett’s test of sphericity supported the scale’s suitability for use with Arabic-speaking populations.

### A nursing competence assessment tool among nursing students

The Nurse Competence Scale, developed by Huang et al. (2022), was chosen to assess the comprehensive nursing competence of students [35]. The scale contains 30 items distributed across six subscales: Medical-related Knowledge, Basic Nursing Skills, Communication and Cooperation, Lifelong Learning, Global Vision, and Critical Thinking. Participants rate each item on a five-point Likert scale from 1 (Strongly Disagree) to 5 (Strongly Agree), resulting in a total score range of 30 to 150. A higher total score indicates higher competence in nursing. For this study, the scores were interpreted as follows: low competence (30–69), moderate competence (70–109), and high competence (110–150). This categorization allows for a clearer understanding of the students’ competencies in various aspects of nursing practice. The Arabic version of the scale exhibited strong reliability with a Cronbach’s alpha of 0.954. The content validity was confirmed through exploratory factor analysis, which explained 80.35% of the total variance, and the KMO and Bartlett’s test indicated that the data were suitable for factor analysis.

### Student time management scale (STMS)

The Student Time Management Scale (STMS), developed by Balamurugan (2013), was employed to measure students’ time management skills [36]. The scale consists of 28 items grouped into four subscales: Scheduling and Prioritizing, Planning and Goal Setting, Reviewing and Record Keeping, and Organizing and Controlling. Each item is rated on a six-point Likert scale from 1 (Strongly Disagree) to 6 (Strongly Agree), with total scores ranging from 28 to 168. Higher scores on this scale indicate better time management skills. The scores were categorized into three levels: poor time management (28–74), moderate time management (75–121), and good time management (122–168). This classification enables the distinction of students’ time management abilities in a way that reflects real-world application. In the present study, the Arabic version of the STMS demonstrated strong reliability, with a Cronbach’s alpha of 0.89. The validity of the scale was further supported through exploratory factor analysis, which explained 78.15% of the total variance. The KMO measure and Bartlett’s test of sphericity affirmed the scale’s suitability for factor analysis in Arabic-speaking populations.

### Study procedures

#### Tool Preparation and pilot study

Permission to use the instruments was obtained via email from the original authors.

The research instruments, including the Educational Leadership Scale for Nursing Students, the Nursing Competence Assessment Tool for Nursing Students, and the Student Time Management Scale, were meticulously translated into Arabic. Bilingual experts fluent in both English and Arabic translated to ensure accuracy and cultural appropriateness. A back-translation into English was performed to verify the translations, ensuring linguistic equivalence and resolving any discrepancies. Following the translation and back-translation process, face validity was assessed by expert panels who reviewed the instruments to confirm that they accurately reflected the intended constructs within the Arabic context. Additionally, feedback was gathered from potential participants to ensure the clarity, relevance, and cultural suitability of the translated items. Reliability was evaluated using statistical methods, including Cronbach’s alpha, to confirm internal consistency. A pilot study involving 50 students was conducted to test the instruments’ clarity, relevance, and reliability. These participants were excluded from the main study. The pilot study results indicated no modifications were needed, confirming the instruments’ suitability for the primary research.

To ensure confidentiality, participants were informed that their responses would remain anonymous and only be used for research purposes. Data were securely stored with restricted access, and identifying information was not collected. Participants were assured that their participation was voluntary, and they could withdraw at any time without consequence. Data analysis was conducted in a manner that ensured individual responses could not be traced back to specific participants.

### Data collection

Data collection for this study was conducted from September to October 2024. The process commenced after obtaining the necessary permissions and securing Excel spreadsheets containing the details of all undergraduate students from the academic affairs department. Participants were selected using a random number generator, repeating the process until the required number of students from each academic year was achieved. Before data collection, researchers gave each student a detailed explanation of the study’s objectives, emphasizing voluntary participation. Written informed consent was obtained from all participants as a prerequisite for their involvement. Researchers assured participants that their responses would remain confidential to ensure confidentiality and foster trust. Questionnaires were distributed in quiet locations, such as empty lecture halls and libraries, between 9 am and 2 pm from Saturday to Thursday. On average, participants spent 15 to 20 min completing each questionnaire.

### Data analysis

Data analysis was performed using SPSS 26.0 (IBM et al., USA) to examine the survey responses from the 532 nursing students. Descriptive statistics, including frequencies (No/%) and mean ± standard deviations (S.D.), were utilized to summarize the participants’ general characteristics and the scores obtained on various scales: t-test and ANOVA test were used to measure the relation between variables and participants’ data. Pearson’s correlation analysis evaluated correlations between nursing students’ educational leadership, time management, and competencies. The analysis of moment structures (AMOS) Version 26 was used to test the mediating role of educational leadership in terms of time management and competencies. Overall Model Fit Criteria (in SEM): These indices evaluate how well the proposed model reproduces the observed data: Chi-Square Test (χ²): Tests the difference between observed and model-implied covariance matrices. *P* > .05 suggests good fit, but it is sensitive to sample size (often significant with large samples even when fit is acceptable). Comparative Fit Index (CFI): Compares your model to a null (independence) model. CFI ≥ 0.95 indicates excellent fit, ≥ 0.90 acceptable. Tucker-Lewis Index (TLI). Similar to CFI but penalizes model complexity. TLI ≥ 0.95 preferred. Root Mean Square Error of Approximation (RMSEA): Penalizes lack of parsimony. RMSEA ≤ 0.06 (excellent), **≤** 0.08 (acceptable). Standardized Root Mean Square Residual (SRMR): Measures the standardized difference between observed and predicted correlations.SRMR ≤ 0.08 indicates a good fit.

## Results

A total of 523 nursing students participated in the survey. Less than half, 46.8% of the participants were younger than 20, and more than half, 59.2%, were female. Also, study involved 25% of all academic year 25% were in their second, 72.6% didn’t work, 78% were from urban areas, about two thirds 64.1% had enough monthly income, 38.2% of their mothers had secondary education, and less than half 43.8% of their fathers had secondary education Table 1.

**Table 1:** Personal characteristics among participants (*n* = 532)

Personal Characteristics	Categories	No	%
Age	Less than 20	249	**46.8**
	From 20 to less than 22	236	44.4
	22 years and more	47	8.8
Gender	Male	217	40.8
	Female	315	**59.2**
Academic year	First	133	25.0
	Second	133	25.0
	Third	133	25.0
	Fourth	133	25.0
Occupation	Not working	386	**72.6**
	Working	146	27.4
Place of residence	Rural	117	22.0
	Urban	415	**78.0**
Family type	Nuclear	340	63.9
	Extended	192	36.1
Family income	Enough	341	64.1
	Enough & Save	72	13.5
	Not Enough	119	22.4
Mother’s educational level	Basic education	164	30.8
	Secondary education	203	38.2
	University & above	165	31.0
Father’s Educational Level	Basic education	146	27.4
	Secondary education	233	43.8
	University & above	153	28.8

Regarding descriptive study variables, the mean score of the participants’ educational leadership was 78.712 ± 8.721, with subscales: Visionary Leadership was 41.514 ± 4.711, Instructional Leadership was 20.448 ± 2.879, and Scientific Leadership was 16.755 ± 2.247. Regarding Nursing students’ competencies, the total mean score was 117.260 ± 16.019; with subscales, Medical related knowledge was 18.471 ± 3.408, Basic nursing skills was 20.109 ± 2.843, Communication and cooperation was 20.093 ± 3.001, Life-long learning was 19.685 ± 3.014, Global vision was 19.298 ± 3.324, and Critical thinking was 19.603 ± 2.953. Also, the mean score for time management was 119.703 ± 15. 388 Table 2.

**Table 2:** Descriptive and correlational analysis between study variables (*n* = 532)

Variables	Mean ± SD	1	2	3	4	5	6	7	8	9	10	11	12
Visionary Leadership (1)	41.514 ± 4.711	1	0.763^**^	0.585^**^	0.943^**^	0.309^**^	0.445^**^	0.449^**^	0.418^**^	0.348^**^	0.425^**^	0.458^**^	0.204^**^
Instructional Leadership (2)	20.448 ± 2.879			0.574^**^	0.891^**^	0.250^**^	0.376^**^	0.390^**^	0.373^**^	0.318^**^	0.391^**^	0.401^**^	0.212^**^
Scientific Leadership (3)	16.755 ± 2.247			1	0.763^**^	0.329^**^	0.444^**^	0.441^**^	0.497^**^	0.403^**^	0.403^**^	0.483^**^	0.225^**^
**Educational Leadership (4)**	78.712 ± 8.721				1	0.334^**^	0.479^**^	0.485^**^	0.477^**^	0.397^**^	0.463^**^	0.504^**^	0.238^**^
Medical-related knowledge (5)	18.471 ± 3.408					1	0.665^**^	0.654^**^	0.604^**^	0.659^**^	0.622^**^	0.818^**^	0.280^**^
Basic nursing skills (6)	20.109 ± 2.843						1	0.823^**^	0.708^**^	0.600^**^	0.632^**^	0.847^**^	0.338^**^
Communication and cooperation (7)	20.093 ± 3.001							1	0.765^**^	0.728^**^	0.691^**^	0.895^**^	0.387^**^
Life-long learning (8)	19.685 ± 3.014								1	0.771^**^	0.743^**^	0.883^**^	0.336^**^
Global vision (9)	19.298 ± 3.324									1	0.790^**^	0.881^**^	0.363^**^
Critical thinking (10)	19.603 ± 2.953										1	0.862^**^	0.346^**^
**Competence (11)**	117.260 ± 16.019											1	0.394^**^
**Time management (12)**	119.703 ± 15.388												

Furthermore, the study findings clarify a statistically significant positive correlation between nursing students educational leadership and competencies at (*r* = .504^**^, *P* < .001), and nursing students educational leadership and time management at (*r* = .238^**^, *P* < .001), and nursing students competencies and time management at (*r* = .394^**^, *P* < .001) Table 2.

Regarding the relationship between total study variables and studied nurses’ characteristics, there was a statistically significant difference between the participants’ age and educational leadership and competencies (*P* = .028 & <0.001) high mean related to age from 20 to less than 22 years old, with 80.3404 ± 7.616 and 126.6596 ± 13.316, respectively. Also, there was a statistically significant difference between the participants’ academic years and nursing students’ competencies at (*P* = .026), with a high mean related to fourth-year students with 121.1231 ± 14.91. Regarding family income, there was a statistically significant difference between the participants’ family income and educational leadership, competencies, and time management, with a high mean related to enough income at (*P* = .003, < 0.001 & <0.001) with 79.1988 ± 9.108, 119.5513 ± 16.361& 124.5294 ± 10.254 respectively Table 3.

**Table 3:** Relationship between total study variables and studied nurses’ characteristics (*n* = 532)

Personal Characteristics	Educational leadership		Competences		Time management	
	M (SD)	t/F (*P*)	M (SD)	t/F (*P*)	M (SD)	t/F (*P*)
**Age**						
Less than 20	77.6811 ± 8.61	**3.591** **0.028** ^*^	114.4055 ± 17.328	**13.378** **< 0.001** ^**^	119.1024 ± 16.876	1.512 0.221
From 20 to less than 22	79.5000 ± 8.938		118.4619 ± 14.121		119.6271 ± 13.5748	
22 years and more	80.3404 ± 7.616		126.6596 ± 13.316		123.3404 ± 15.3541	
Gender						
Male	78.0000 ± 9.725	1.581 0.115	116.8864 ± 18.44	− 0.451 0.652	119.3318 ± 18.28	0.466 0.667
Female	79.2082 ± 7.929		117.5205 ± 14.115		119.9621 ± 13.028	
**Academic year**						
First	76.8793 ± 10.387	1.869 0.134	118.5345 ± 21.446	**3.100** **0.026** ^**^	121.9138 ± 20.4813	0.878 0.452
Second	78.3818 ± 8.014		115.3491 ± 15.84		119.8655 ± 14.623	
Third	79.5468 ± 9.478		118.7050 ± 13.721		118.2014 ± 14.70	
Fourth	79.9692 ± 8.102		121.1231 ± 14.91		120.2615 ± 14.758	
**Occupation**						
Not working	78.8355 ± 8.447	0.526 0.599	117.5167 ± 15.72	0.600 0.549	120.1877 ± 14.654	1.181 0.561
Working	78.3919 ± 9.4268		116.5878 ± 16.802		118.4324 ± 17.156	
**Place of residence**						
Rural	79.1008 ± 8.674	0.549 0.583	119.7983 ± 16.9293	1.964 0.050	121.2521 ± 16.123	1.245 0.061
Urban	78.6029 ± 8.742		116.5383 ± 15.697		119.2632 ± 15.163	
**Family type**						
Nuclear	79.1953 ± 8.514	1.706 0.890	117.6560 ± 15.854	0.760 0.962	119.1633 ± 15.113	1.083 0.428
Extended	77.8608 ± 9.035		116.5619 ± 16.325		120.6598 ± 15.856	
**Family income**						
Enough	79.6540 ± 8.857	**5.908** **0.003** ^**^	119.5513 ± 16.361	**14.089** **< 0.001** ^**^	124.5294 ± 10.254	**75.490** **< 0.001** ^**^
Enough & Save	77.7403 ± 10.57		109.3117 ± 20.074		121.9619 ± 13.40	
Not Enough	76.6471 ± 6.321		115.8403 ± 8.741		102.2468 ± 18.310	
**Mother’s educational level**						
Basic education	79.1988 ± 9.108	1.293 0.275	116.8916 ± 16.274	0.421 0.657	118.6627 ± 14.821	1.659 0.191
Secondary education	77.9466 ± 8.649		116.7961 ± 15.3001		119.1068 ± 15.38	
university & above	79.1818 ± 8.392		118.2121 ± 16.683		121.4970 ± 15.879	
**Father’s Educational Level**						
Basic education	78.0342 ± 8.796	0.725 0.485	117.7466 ± 14.73	1.888 0.152	119.1027 ± 13.182	0.446 0.640
Secondary education	79.1392 ± 8.386		115.8439 ± 16.66		119.4473 ± 15.771	
University & above	78.7013 ± 9.161		118.9805 ± 16.08		120.6688 ± 16.72	

**Table 4:** Mediating effect of educational leadership between time management and nursing students’ competences (*n* = 532)

Direct effect	(B)	CI 95%	T	*p*
Time management → Educational leadership	0.411	(0.380–0.596)	8.895	< 0.001^**^
Time management → Competences	0.024	(0.193–0.423)	5.260	< 0.001^**^
Educational leadership → Competences	0.48	(0.250-0.433)	7.355	< 0.001^**^
**Indirect effect**				
Time management → Educational leadership→ Competences	0.567	(0.101–0.233)	4.968	< 0.001^**^
**Total effect**				
Time management → Competences	0.485	(0.366–0.584)	8.554	< 0.001^**^

**Fig. 2:**
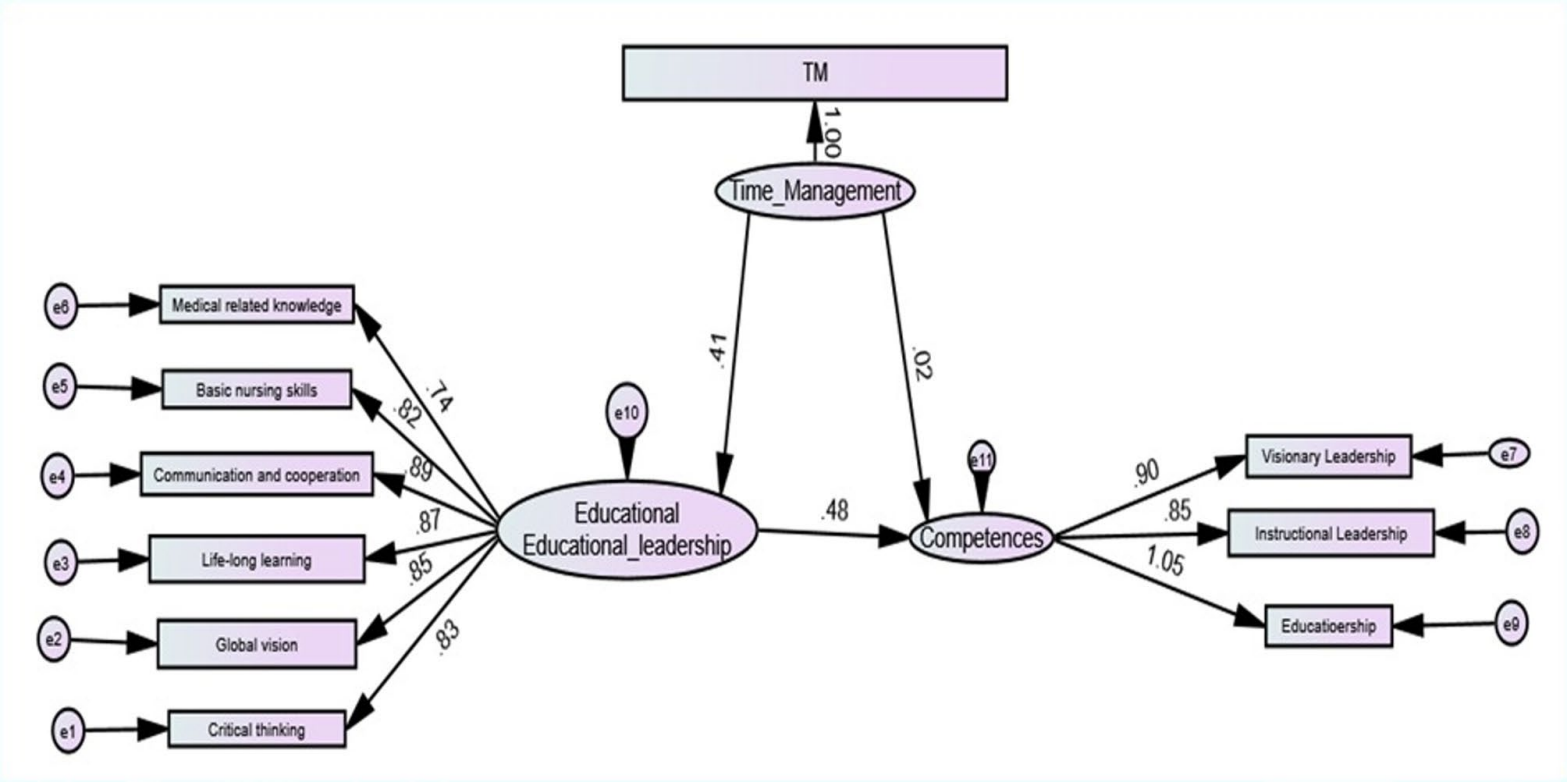
Mediation model

As show in Table (4) and figure (2) there was a significant direct effect of time management on educational leadership at (B = 0.411, t = 8.895, P = < 0.001), time management on competencies (B = 0.024, t = 5.260, *P* < .001) and between educational leadership on competencies (B = 0.48, t = 7.355 *P* < .001). Furthermore, there was a significant indirect effect of time management as an independent variable on competences as the dependent variable in the presence of educational leadership as a mediator variable (B = 0.567, 4.968, *P* < .001).

## Discussion

This study explored how time management and educational leadership relate to nursing competency among undergraduate students, with a particular focus on the mediating role of educational leadership. The findings contribute meaningfully to the current literature by emphasizing how personal and institutional factors intersect to shape student performance in nursing education.

### Time management and nursing competency

The results confirmed that time management plays a vital role in developing nursing competencies. Students with stronger time management skills were better able to balance academic responsibilities, clinical rotations, and personal commitments, ultimately leading to higher competency scores. These findings align with existing literature, which has highlighted time management as a significant predictor of academic performance and clinical readiness [37–39]. Structured time allocation enables nursing students to stay organized, prioritize tasks, and engage more meaningfully with clinical learning opportunities, as reflected in previous studies showing similar outcomes among students and professional nurses [40]. This highlights that nursing programs should intentionally embed time management training into the curriculum, especially in early academic phases, to reinforce clinical preparedness and cognitive flexibility.

Notably, our findings extend this understanding by demonstrating that time management does not operate in isolation. While earlier work has recognized its value, some scholars have cautioned that its effects may be limited without supporting structures such as feedback mechanisms, goal setting, and institutional reinforcement [20, 23]. In our study, time management had a more substantial impact on competency when students also reported higher levels of educational leadership.

This suggests that effective time use requires an enabling environment where students are guided, mentored, and supported by academic leaders. Without this support, the benefits of time management may be reduced to mere task completion rather than meaningful competency development. Therefore, time management education in nursing programs should be accompanied by regular mentorship, structured feedback, and practical scheduling tools to help students internalize these skills in both academic and clinical settings [41].

### Educational leadership and competency

In addition to time management, educational leadership emerged as a significant and independent contributor to nursing competency. Students who perceived their educators as supportive, organized, and inspirational consistently reported higher confidence and skill proficiency. This finding supports prior research highlighting the value of visionary and instructional leadership in healthcare education [18, 25, 42]. Academic leaders who model reflective practice, encourage student autonomy, and cultivate critical thinking foster environments where students can thrive both cognitively and emotionally. However, our findings also offer a distinctive perspective by suggesting that leadership becomes most effective when paired with structured, real-world applications.

While theoretical leadership content is important, its impact on clinical competence is amplified when integrated with experiential learning, such as simulation, mentorship, and peer collaboration [26]. This distinction is particularly important in systems where educational leadership is sometimes limited to classroom instruction rather than immersive student engagement. These findings imply that leadership development initiatives should not be confined to theory but need to be embedded in hands-on clinical teaching and mentorship, enhancing students’ readiness for professional roles.

Although the role of leadership is widely recognized, some studies challenge the extent of its influence, noting that individual characteristics—such as motivation, emotional intelligence, and resilience—may play a more direct role in competency development than external leadership [43, 44]. While our study acknowledges the influence of such internal factors, the findings demonstrate that educational leadership can strengthen and direct students’ use of personal skills. By shaping the learning climate, reinforcing time management behaviors, and setting clear expectations, educational leaders enhance the effectiveness of student-led efforts.

The ability to manage time, for example, becomes more impactful when reinforced by leaders who provide scheduling guidance, academic prioritization strategies, and performance-based feedback [27]. This finding underscores the importance of viewing leadership not as an isolated institutional trait but as an active force that nurtures student autonomy and competence. From an institutional perspective, this calls for structured faculty development programs that equip educators to actively mentor, coach, and model leadership and time efficiency behaviors for students.

### The mediating role of educational leadership

A central contribution of this study lies in the identification of educational leadership as a mediating factor between time management and competency. This mediation implies that leadership enables students to translate organizational abilities into actual clinical performance. Strong leadership environments create the structural and motivational conditions necessary for students to apply their time effectively and build practical skills. The findings align with prior literature suggesting that academic leaders influence learning through guidance, mentorship, and behavioral modeling [44, 25]. Leaders who embody professional values and model time-conscious behavior enable students to integrate those habits into their routines, fostering greater readiness for clinical demands. These outcomes not only support the educational process but also reflect broader institutional objectives related to patient safety, communication, and quality care.

However, it is important to acknowledge that not all findings in the literature support the central role of leadership. Some studies argue that leadership alone cannot fully compensate for a lack of student motivation or inadequate self-regulation [45–47]. Our results suggest that educational leadership can maximize the benefits of time management, but its influence may be constrained in settings where institutional resources are limited or where faculty are not trained in effective leadership strategies. Furthermore, cultural and contextual differences may moderate these effects. In some academic settings, students are encouraged to be highly autonomous, and leadership is perceived as distant or hierarchical. In contrast, in more collaborative environments, students may rely heavily on educator guidance [48]. These contextual differences help explain why leadership may be more influential in some studies than others. Therefore, educational strategies should be culturally adapted and resource-aware, recognizing how leadership dynamics vary by institutional environment.

The implications of these findings are multifaceted. From a curricular perspective, nursing programs should integrate leadership development and time management training into foundational coursework. Educational interventions should be designed to help students build not only task-planning skills but also an understanding of how to apply those skills in complex, high-stakes environments. Leadership development for educators is equally important. Training that equips academic staff to model time-conscious behavior, foster student engagement, and mentor students toward professional growth can create cascading benefits throughout the educational system. Additionally, academic institutions should consider embedding reflective practice, simulation-based learning, and peer-led leadership opportunities to reinforce the synergy between time management and competency [2, 5, 48].

This study offers valuable insights for broader applications beyond nursing. Other healthcare disciplines, and even non-health-related academic fields, may benefit from the integration of leadership and time management into competency frameworks. In professions that demand high levels of coordination, responsibility, and skill precision, the interplay between individual time use and institutional leadership is equally relevant. Policies that support leadership development, foster a culture of structured learning, and prioritize mentoring are likely to see improved outcomes in student confidence, performance, and long-term professional preparedness.

## Conclusion

This study reveals the significant role of educational leadership as a mediator in the relationship between time management and nursing students’ competence. The results highlight that effective time management directly improves educational leadership and competencies. Additionally, educational leadership further enhances the competencies of nursing students. This finding underscores the importance of leadership qualities in fostering essential nursing skills and the need for educational programs to emphasize leadership development. The study also shows that age, family income, and academic year significantly influence students’ competencies and leadership. This study emphasizes that nurturing educational leadership can bridge the gap between effective time management and enhanced competence among future nurses. Investing in leadership development programs within nursing education may lead to more competent, well-prepared healthcare professionals.

### Implications for nursing education, research, and practice

In nursing education, the findings highlight the critical role of time management and educational leadership in enhancing student competencies. Nursing curricula should prioritize the integration of leadership development and time management skills into both theoretical coursework and clinical practice. To achieve this, actionable leadership training strategies could include the implementation of structured leadership workshops and seminars that focus on key competencies such as communication, decision-making, conflict resolution, and team management. Additionally, mentorship programs can be established, where nursing students are paired with experienced nurse leaders to gain hands-on insights and guidance, allowing them to develop practical leadership skills in real-world settings. Simulations and role-playing exercises could also be incorporated into the curriculum, providing students with opportunities to practice managing teams and making decisions under pressure. Moreover, incorporating competency-based assessments of leadership abilities into nursing programs ensures that students are continually evaluated and given constructive feedback for improvement. Interdisciplinary collaboration exercises, where students are encouraged to work with peers from other healthcare disciplines, could also help strengthen leadership skills and foster teamwork.

To implement these strategies effectively, nursing institutions should embed leadership training into the core nursing curriculum through mandatory courses and clinical placements. Furthermore, providing continuous professional development programs for faculty members will ensure that they are equipped with the necessary skills and knowledge to model and teach effective leadership. In doing so, institutions can build a strong leadership foundation that prepares nursing students to navigate the complexities of the healthcare environment and excel in their professional roles.

For nursing research, this study opens avenues for further investigation into the relationships between leadership, time management, and student competencies. Future research could explore the long-term impact of leadership training and time management skills on nursing students’ professional practice, including clinical decision-making, patient care, and interdisciplinary collaboration. Longitudinal studies could track the outcomes of leadership training programs, examining how these skills influence nursing students’ career trajectories and their ability to manage the demands of the nursing profession. Additionally, comparative studies across different nursing programs and educational contexts could shed light on the most effective leadership training methods and their impact on student competencies. Research focusing on technology-driven leadership training and its role in preparing nursing students for the digital healthcare landscape would also be valuable. Investigating these gaps in the literature will provide deeper insights into how leadership and time management influence nursing practice and contribute to improved healthcare outcomes.

### Strengths and limitations

One of the key strengths of this study is the use of a well-validated set of tools to assess educational leadership, time management, and nursing competency. The inclusion of robust scales, such as the Educational Leadership Scale for Nursing Students, the Nurse Competence Scale, and the Student Time Management Scale, ensures that the measured constructs are reliable and valid, enhancing the credibility of the findings. Additionally, the translation and validation process for these scales into Arabic further strengthens the study’s relevance and applicability to Arabic-speaking populations, allowing for culturally appropriate assessments. Another strength is the use of a systematic random sampling method, which helps to reduce selection bias and ensures balanced representation across all academic years. The relatively large sample size also contributes to the robustness of the results, providing sufficient statistical power to detect meaningful relationships between educational leadership, time management, and competency. The pilot study conducted before the primary research helped ensure the instruments’ clarity and reliability, reducing the possibility of misinterpretation and enhancing the study’s internal consistency.

Despite its strengths, the study has several limitations. First, the study was conducted within a single institution, which limits the generalizability of the findings. Since the data were collected from students at one university, the results may not reflect the experiences or competencies of nursing students at other institutions with different educational structures, curricula, or student populations. This limitation may restrict the extent to which the findings can be applied to nursing schools in other geographic locations or cultural contexts. To enhance the generalizability of the results, future studies could expand the sample size to include multiple institutions from diverse regions or countries, offering a more comprehensive understanding of the relationship between leadership, time management, and competency across varied settings.

Another limitation is the reliance on self-reported data, which may introduce biases such as social desirability or inaccurate recall. Although validated scales were employed in this study, self-report measures are still subject to participants’ perceptions and memory recall, which may not always be accurate. As a result, the findings may not fully reflect participants’ true behaviors, attitudes, or competencies. Future studies could address this limitation by incorporating objective measures, such as academic performance records, practical skill assessments, or peer evaluations, to provide a more accurate and holistic assessment of students’ competencies and leadership abilities.

Additionally, the cross-sectional design of the study limits the ability to establish causal relationships between the variables. As data were collected at a single point in time, it is unclear whether leadership qualities, time management skills, and competency are the cause or consequence of one another. Longitudinal research would be beneficial in this area, as it would allow for the exploration of how these factors evolve and their potential impact on students’ future professional performance.

## Data Availability

The datasets generated and analyzed during the current study are not publicly available due to confidentiality agreements but are available upon reasonable request from the corresponding author.
